# Establishment and characterization of novel human primary endometrial cancer cell line (ZJB-ENC1) and its genomic characteristic

**DOI:** 10.7150/jca.33013

**Published:** 2019-10-20

**Authors:** Xiaozhen Liu, Zhuozhuo Ren, Yu Xu, Wei Sun, Yongfeng Li, Xinmiao Rui, Dafei Xie, Xuli Meng, Zhiguo Zheng

**Affiliations:** 1 Institute of Cancer and Basic Medicine (ICBM), Chinese Academy of Sciences, Hangzhou, 310022, China; 2 The Experimental Center, Cancer Hospital of the University of Chinese Academy of Sciences, Hangzhou, 310022, China; 3 The Experimental Center, Zhejiang cancer hospital , Hangzhou, 310022, China; 4 Medical Support Department, Zhejiang Provincial Hospital of Traditional Chinese Medicine, Hangzhou 310022, China; 5 Department of Hematology, The First Affiliated Hospital, Zhejiang University, School of Medicine, Hangzhou, 310022, China; 6 Department of Breast Surgery, Cancer Hospital of the University of Chinese Academy of Sciences, Hangzhou, 310022, China; 7 Department of Breast Surgery, Zhejiang cancer hospital, Hangzhou, 310022, China; 8 The Second Clinical Department, Zhejiang Chinese Medical University, Hangzhou, 310022, China; 9 General Surgery Department, Zhejiang Hospital, Hangzhou, 310022, China; 10 Department of Breast Thyroid Surgery, Tongde Hospital of Zhejiang Province, Hangzhou, 310022, China

**Keywords:** Endometrioid adenocarcinoma, Cell line, Tumorigenicity, Immunohistochemisty, Whole exome sequencing

## Abstract

The establishment of human malignant tumor cell lines can provide abundant experimental materials for understanding the biological characteristics of tumors, studying the carcinogenesis, molecular genetics and the mechanism of metastasis and evolution. In this study, a novel cell line designated ZJB-ENC1 has been established from poorly differentiated endometrioid adenocarcinoma. Cytological results showed monolayer-cultured cells were polygonal in shape and a piling-up tendency without contact inhabitation. Immunohistochemistry analysis showed that the cells were negative for *ER*, *PR*, *c-erbB2*, *E-CAD*, *CD117*, and *OCT3/4*, but strongly positive for *PTEN* and *P16*. Meanwhile, the tumorigenicity of ZJB-ENC1 was confirmed by subcutaneous transplantation of the cells into a xenograft mouse model. In addition, the results of the whole exome sequencing revealed a unique genomic characteristic of ZJB-ENC1 cells, all common and novel SNPs and InDels were identified. In conclusion, this new stable cell line may promote basic and clinical research on endometrial cancer (EC).

## Introduction

Endometrial carcinomas (EC) is a malignant epithelial tumor that arises from the endometrium owing to the precursor lesions such as complex hyperplasia with atypia^1^. It is a common malignancy of female genital tract and the third most common cancer in women^1^. The first sure symptom of EC is often atypical genital bleeding not associated with menstrual period. The most frequent type of EC is endometrioid carcinoma, which counts for more than 80% of cases^2^. The incidence and mortality rate of EC are gradually increasing, according to reports, the incidence rate per 100,000 women is 12.9 in more developed regions^3^. Approximately 52,630 new cases had been diagnosed in 2014 and the annual monitoring of cancer deaths reported almost 8590 events each year in the United States (US)^4^. Only in 2008, in the European countries, 12903 women died from EC, and corrected age-standardized mortality rates have decreased significantly over the past decades in most member states^5^. The increasing incidence of morbidity suggests that the pathogenesis of the disease is an urgent problem to be solved.

Cell lines of endometrial origin may provide useful tools to study the biology of the disease and to develop and test novel therapeutic approaches. A large bank of well-characterized cell lines should reflect the diversity of tumor phenotypes and provide adequate models for the study of tumor heterogeneity. Additionally, disease-orientated drug screening using human tumor cell lines *in vitro* has some predictive value for the activity of clinical responses^6^, ^7^. To date, despite a number of endometrial carcinoma cell lines have been reported^8^-^15^, few originated from poorly differentiated endometrioid adenocarcinoma. Therefore, it is absolutely necessary to establish stable and available cell lines of endometrioid adenocarcinoma.

The advent of massively sequencing technologies has markedly expanded knowledge of genome-wide gene abnormalities in various tumors. The whole exome sequencing (WES) is a genomic technique for sequencing all of the protein-coding genes in a genome, which consists of capturing and sequencing of exome. It has been widely used to characterize the mutational spectrum of various cancers^16^-^18^ and provide amount of genetic information for further study.

In this paper, a novel EC cell line ZJB-ENC1 originated from a 58-year-old patient with poorly differentiated endometrioid adenocarcinoma was established and analyzed with respect to the growth property, cellular ultrastructure, neoplastic behavior in SCID nude mice and cell line authentication by short tandem repeat (STR) profiling. Moreover, the mutated genes with known and novel genomic abnormalities were identified by the whole exome sequencing.

## Materials and methods

### Patient

The cell line was derived from an endometrioid adenocarcinoma patient who was a 58-year-old woman in Zhejiang Cancer Hospital. She was treated with curettage in a local hospital and the symptoms were alleviated subsequently. In May 2015, she underwent surgery for the EC because of recurrence. Laboratory examination results showed CA724 13.36 U/ml, CA125 209.40 U/ml and SCC 2.0 ng/ml. The resected tumor was approximately 2.5×2.3×0.8 cm, pathological results showed moderately poorly differentiated endometrioid adenocarcinoma with chronic inflammation of 18 lymph nodes. The written informed consent was obtained from the patients, which was approved by the Ethical Committees of Zhejiang Cancer Hospital, Hangzhou, China.

### Establishment of ZJB-ENC1 cell line

EC tissue was obtained during surgery from the patient and immediately processed. Specimens were washed with RPMI medium supplemented with 10% FBS, 100 U/ml penicillin and 100 µg/ml streptomycin and minced into small pieces. Pieces were digested with a mixed enzyme (V_trypsin-EDTA_ : V_type II collagenase_ = 1:1) for 2 hours and filtered by 40 µm cell strainer to remove large fragment. The flow-through was collected by centrifugation. Cancer cells were resuspended and cultured in growth medium (RPMI medium : DMEM/F12 : DMEM=2:2:1, supplemented with 10% FBS, 100 U/ml penicillin, 100 µg/ml streptomycin, 100 nM hydrocortisone) and incubated at 37 ^o^C in a humidified atmosphere with 5% CO_2_. The medium was replaced every 3 days. Four days later, the medium was removed and the cells were washed with PBS. Cancer cells were maintained in growth medium till they grew to 80% confluency. The cells were then trypsinized and sub-cultured. Passages 25-40 performed subsequent characterization and testing.

### Cell proliferation assays

Suspension of 1×10^3^ logarithmic phase cells was seeded in 96-well plates in triplicate and cultured in the growth medium. The number of cells was counted daily for 8 days using the Cell Counting Kit-8 (Dojindo, Tokyo, Japan) referring to the instructions by measuring the absorbance at 450 nm at the indicated time-points.

### Short tandem repeat (STR) analysis

Genomic DNA from ZJB-ENC1 was isolated using genomic extraction kit (Axygen, USA) and amplified by 20-STR amplification protocol. The STR loci and sex gene Amelogenin were detected by an ABI 3730XL Genetic Analyzer. The data were processed using GeneScan and GeneMapperTM ID Software (Invitrgen).

### Tumorigenicity in SCID mice

*In vivo* tumorigenicity of ZJB-ENC1 cell line was assessed based on the ability to form tumors in 50 day-old female nude SCID (severe combined immunodeficiency) (SKXK, China) mice at subcutaneous flank injection sites. A volume of 100 μl was injected in each mouse and consisted of 5×10^6^ cells resuspended in 100 μl of cold phosphate buffered saline (D-PBS) (Thermo Fisher Scientific, Waltham, MA, USA). The animals were housed under sterile conditions in a laminar flow environment with unrestricted access to food and water. Tumor formation was observed on Tuesday and Friday for 35 days. The mice were sacrificed and tumors were removed for H&E staining and pathology examination. All animal studies were performed according to protocols approved by the Institutional Animal Study Committee of Zhejiang University of Traditional Chinese Medicine Animal Testing Center.

### Immunohistochemistry

Immunostaining was performed using the Mouse PV Two-Step immunohistochemistry Kit (Beijing ZhongSuanJinQiao Biotechnology Co., LTD., pv-6002). ZJB-ENC1 cells and tumor tissue were processed into paraffin block which could be used for making histological sections. The sections were baked at 60 ^o^C for 1 hour, and incubated with 3% hydrogen peroxide for 10 min after xylol deparaffinization. For all biomarkers, the slides were washed with PBS for 2 × 3 min after incubation with respective primary antibody overnight at 4 °C and incubated for one hour with the secondary antibody at room temperature, washed with PBS for 2 × 3 min again. Image acquisition and section evaluation were performed under a light microscope after DAB coloration.

### Whole-exome sequencing (WES)

Genomic DNA was extracted from cells using genomic extraction kit (Axygen, USA) and underwent WES according to the manufacturer's protocols. The exomes were captured using SeqCap EZ Exome V3 (64Mb, Nimblegen, USA) and sequenced on an Illumina HiSeq X Sequencing System (Illumina, San Diego, CA, USA) at Shoudu Technical Service Company (Suzhou, China). All single nucleotide polymorphisms (SNPs) and insertions and deletions (InDels) were identified by Genome Analysis Toolkit v.3.8 (GATK best practices).

## Results

### Growth characteristics and morphology of ZJB-ENC1 cells

**Figure 1:**
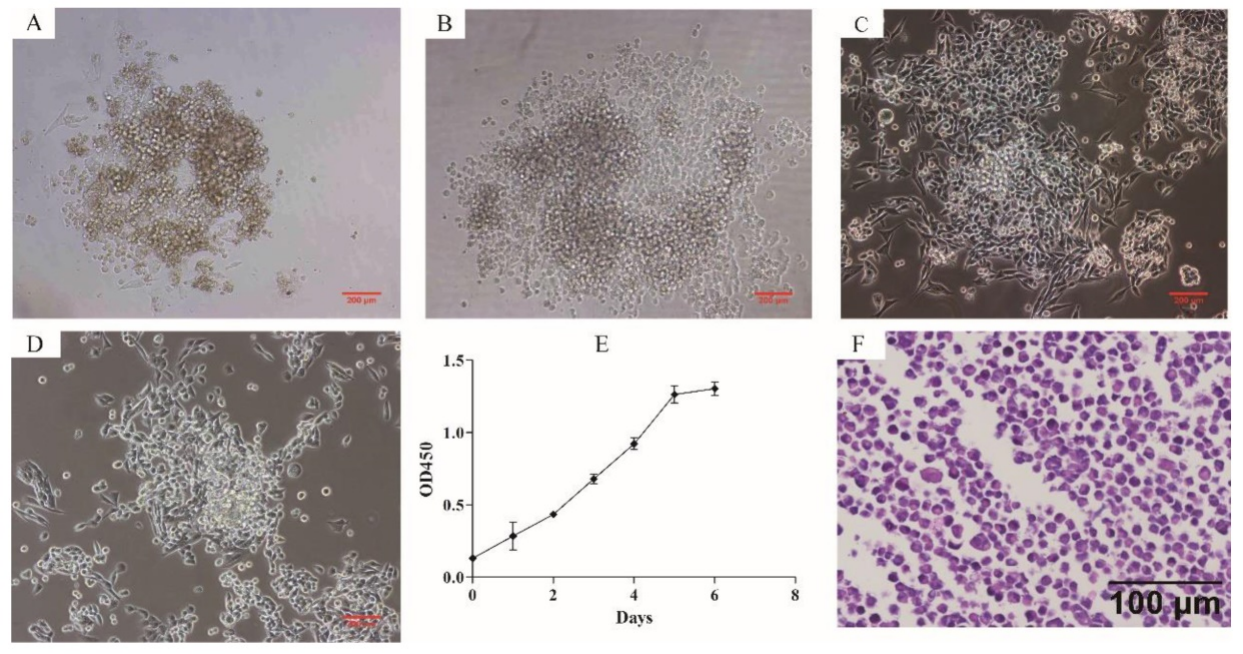
** Cytomorphology and proliferation of cultured ZJB-ENC1 cells. Cells were photographed under microscope at 40 × magnification for passage 0** (A), 7 (B), 17 (C), 50 (D), respectively. (E) The grow curve of ZJB-ENC1 cells. (F) H&E staining of the ZJB-ENC1 cells (Scale bar, 100 µm).

Phase contrast microscope revealed that ZJB-ENC1 cells were anchorage-independent and disorderly grew. Cells tended to formed colonies and pile up upon confluence without obvious touching inhibition, and morphologically most monolayer cells appeared polygonal (Figure 1A-1D). The established cell line was designated as ZJB-ENC1, and cells have been cultured for 2 years (98 passages) with rapid proliferation. The doubling time is approximately 43 hours (Figure 1E). Stained with hematoxylin and eosin, the cells presented polygon-shaped epithelial cells with a high nucleus/cytoplasm ratio and exhibited cellular histomorphology identical to the tumor from the patient (Figure 1F).

### Immunohistochemistry

**Figure 2:**
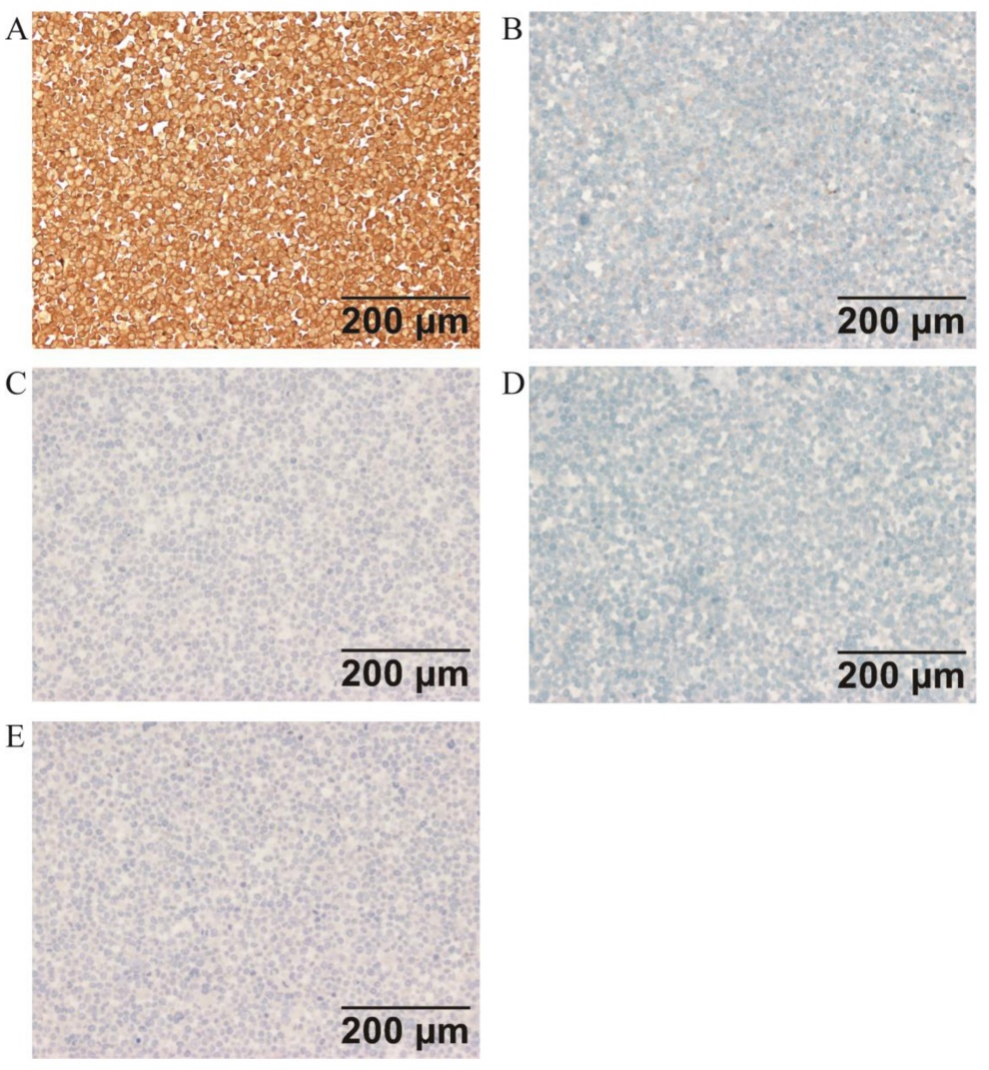
** Immunohistochemical characterization of ZJB-ENC1 cells** (A) multiple tumor suppressor 1 (P16), (B) KIT proto-oncogene, receptor tyrosine kinase (CD117), (C) cadherin 1 (E-CAD), (D) erb-b2 receptor tyrosine kinase 2 (c-erbB2), (E) POU domain, class 5, transcription factor 1 (OCT3/4).

**Figure 3:**
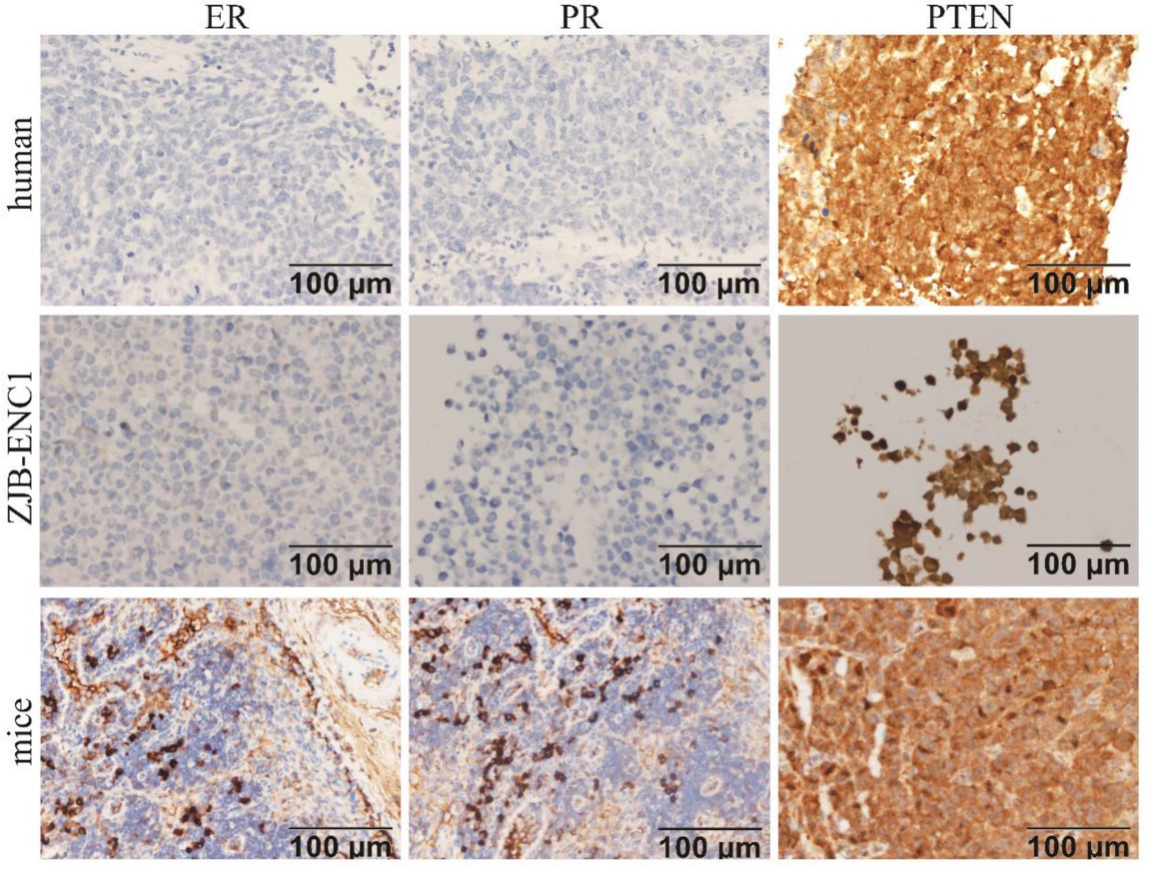
Immunohistochemical characterization of ZJB-ENC1 cells and tumor tissue of mice model and clinical patient. Estrogen receptor (ER), progesterone receptor (PR), phosphatase and tensin homolog deleted on chromosome ten (PTEN) were immunohistochemically evaluated as indicated (Scale bar, 100 µm).

Immunohistochemical analysis of the cell line found that *ER*, *PR*, *c-erbB2*(*HER2*),* E-CAD*, *CD117*, and *OCT3/4* were negative, while *PTEN* and* P16* were strongly expressed in ZJB-ENC1 cells (Figure 2). Additionally, immunohistochemical results of tumor tissue from xenograft mice and the clinical patient showed the same results as ZJB-ENC1 cells with the expression pattern of *ER* (-), *PR* (-) and *PTEN* (+), as illustrated in Figure 3.

### Short tandem repeat (STR)

We performed STR profiling of ZJB-ENC1 cells to avoid the risk of cross-contamination and to confirm it as a novel human EC cell line. STR examination results showed that neither matched sites in the ATCC, DSMZ, JCRB and RIKEN database nor multiple sites existed, indicating that this is a new cell strain and has not been contaminated, as shown in Table 1.

### Tumorigenicity *in vivo*

**Figure 4:**
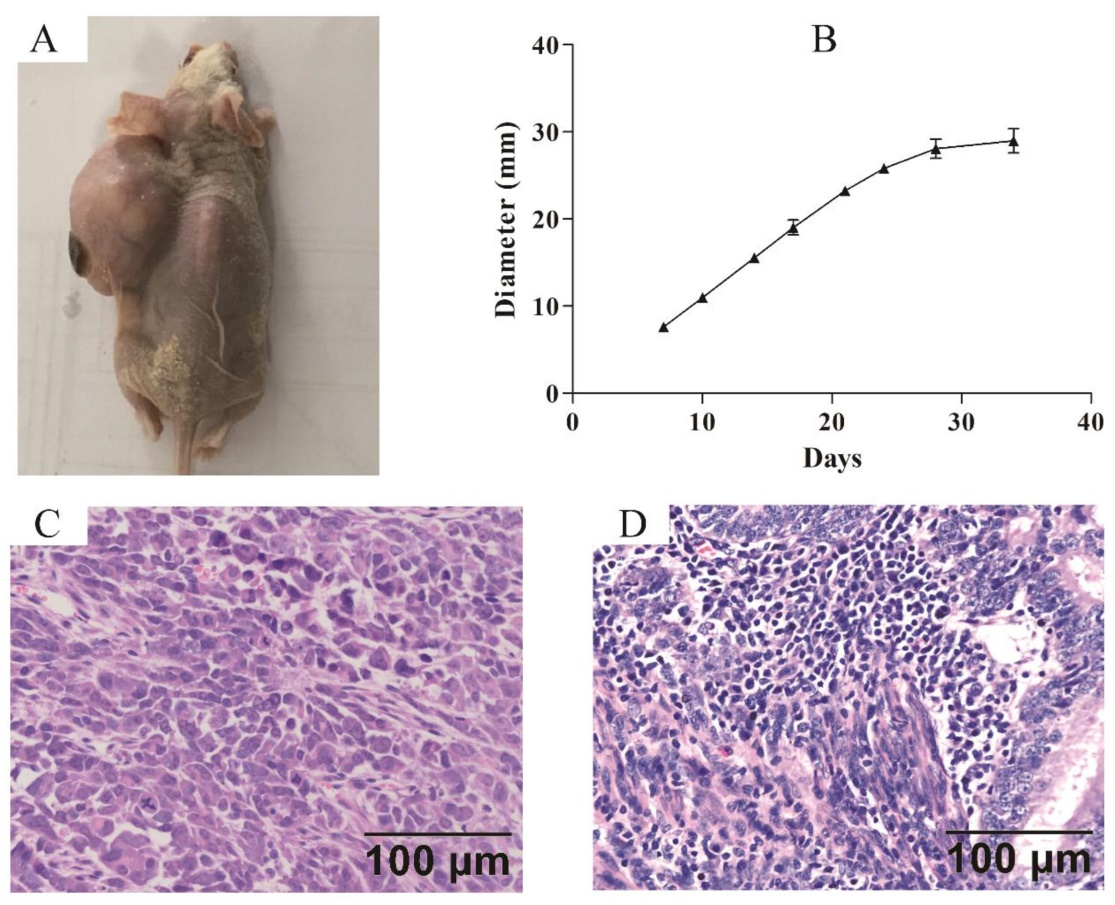
** Nude mouse tumorigenicity assay** (A) The ZJB-ENC1 cells formed tumors at injected site. (B) The tumor growth curve (n = 6), final diameter reached 29 mm after 35 days, tumor size was measured on Tuesday and Friday for 35 days. H&E staining of the tumor specimens in mice (C) and clinical patient (D) (Scale bar, 100µm).

Xenograft model was established to confirm the tumorigenicity of ZJB-ENC1 cells *in vivo*. Subcutaneous injection of ZJB-ENC1 cells in SCID nude mice all successfully formed tumor at injected sites (Figure 4A). The average tumor diameter of 35 days reached 29 mm (n=6) owing to the efficient proliferation ability of ZJB-ENC1 cells (Figure 4B). As expected, the hematoxylin- and eosin-stained tissue sections of the mice tumor xenografts showed similar histologic features as EC (Figure 4C, 4D).

### The whole exome sequencing

**Figure 5:**
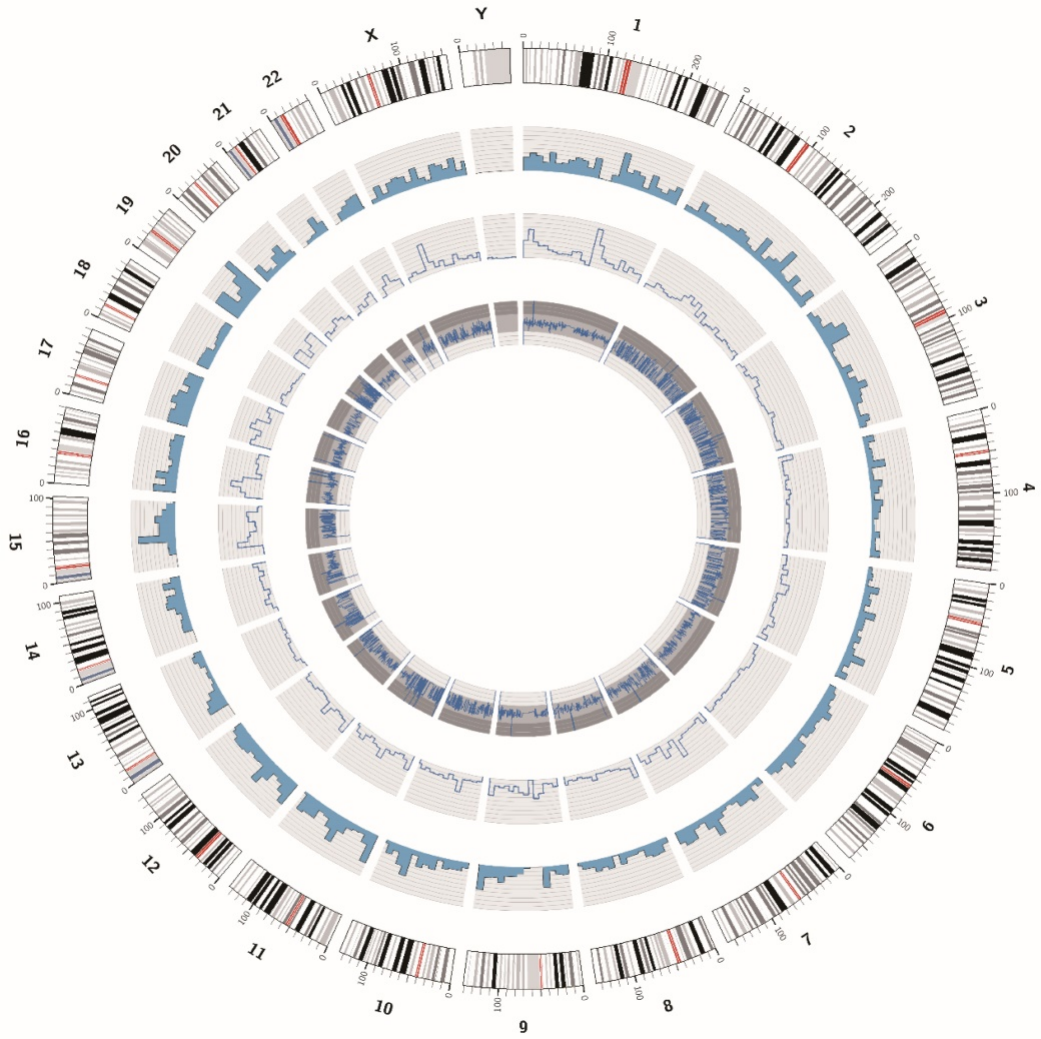
The circos plot of the whole exome sequencing of ZJB-ENC1 cells. Chromosomes are shown in the color coded of the outermost ring. The second ring shows the SNV density. The inner ring represents depth information and the innermost ring shows SNV mutation frequency.

The DNA sample of the ZJB-ENC1 cell line was prepared for the whole exome sequencing. We obtained 129374536 paired raw reads (38.83Gb bases), and the average GC content was 46.44%. The value of Q20 and Q30 were 94.22% and 87.35%, respectively, indicating a high quality of sequencing data. The statistic for the distribution of SNPs and InDels were listed in Table 2. In addition, we showed the circos plot of whole exome sequencing (Figure 5), circles from the outermost to the innermost represented chromosome information, SNV density, depth information and SNV mutation frequency, respectively. Meanwhile, we listed 10 common genes which were proved to be strongly associated with EC, along with their corresponding SNPs and InDels in Table 3.

## Discussion

Despite the cell lines may only present a part of the characteristics of the tumor^19^, it is still necessary to establish novel and well-characterized EC cell lines to investigate the mechanism of genesis and development, and then so as to develop new therapies against cancer. Many adenocarcinoma cell lines derived from human endometrial tissue have been applied to experimental research^8^-^15^. In this literature, we established a new human endometrioid adenocarcinoma cell line, designated ZJB-ENC1. The original tumor of ZJB-ENC1 was histologically diagnosed as a poorly differentiated endometrioid adenocarcinoma. The cell line was well characterized with respect to cell morphology, immunohistochemical and histologic characteristics, growth, oncogenicity. ZJB-ENC1 cells grow rapidly in culture and did not express *ER*, nor *PR*, except for *PTEN*. It was confirmed that the cell line was not contaminated with mycoplasma, bacteria and fungi. The tumorigenesis of ZJB-ENC1 cells was confirmed by the formation of solid tumors in nude mice that injected with ZJB-ENC1 cells. The orthotopic implantation model of ZJB-ENC1 cells can be applied to experimental therapeutics for endometrial carcinoma.

To a great extent, progesterone therapy has been considered to be a favorable treatment for patients with EC^20^, but poorly differentiated and recurrent endometrial carcinomas always show a poor response to progestin owing to the lack of steroid receptors^21^-^23^. Notably, endometrioid adenocarcinoma cell lines are mostly negative for hormone receptor because these cell lines mostly source from poorly differentiated tumors^10^. As we expected, ZJB-ENC1 cells did not express ER nor PR in our study. Furthermore, the ZJB-ENC1 cell line is a triple-negative cell line with absence of ER, PR and c-erbB2 protein expression, which indicates this new cell line of EC may have more comprehensive characteristics and potential value for EC research according to the previous studies of the triple-negative phenotype (TNP) breast cancer^24^.

Previous studies have found that *PTEN* gene abnormalities existed in various cancers, such as prostate cancer^25^, EC^26^, kidney cancer^27^, and so on. It is considered to be another tumor suppressor gene that is closely related to tumorigenesis after the found of P53. Unsurprisingly, a high expression level of PTEN was observed in ZJB-ENC1 cells. Meanwhile, several mutations of *PTEN* were detected by WES, among them, single nucleotide polymorphism (rs701848) carrying the C allele, located on 3-UTR, is associated with increased cancer risk in Asian population^28^. *PTEN* plays a key role in regulating the PI3K-AKT-mTOR signaling pathway by modulating the phosphatidylinositol 3,4,5-trisphosphate^29^, ^30^, as well as maintaining genetic stability caused by double-strand breaks (DSBs)^31^. It suggests ZJB-ENC1 cells is useful for researching the influence of mutations in *PTEN* on endometrioid adenocarcinomas. P16, a tumor suppressor protein encoded by the CDKN2A gene, plays an important role in cell cycle regulation by decelerating the cell's progression from G1 phase to S phase^32^. The polymorphism rs11515 with CG phenotype, locate at the 3'UTR of the CDKN2A gene, has a statistically significant correlation with aggressive breast tumors with decreased p16 (INK4a)^33^. On the contrary, the ZJB-ENC1 cells positively expressed P16 protein with CG phenotype at rs11515, further investigation should be conducted to explain this difference.

WES was applied to obtain the genome-wild mutational landscape of ZJB-ENC1 cells, and the total SNPs and InDels were identified. Meanwhile, we listed the common mutation sites of several genes associated with EC, such as *TP53, KRAS, FGFR2* and *CTNNB1*. TP53 encodes the p53 protein, which is involved in cell cycle regulation, apoptosis, senescence, and DNA repair^34^. The p53 gene is highly polymorphic, with at least 13 different polymorphisms described^35^, ^36^. Among them, p53 intron 3 variant (rs17883323) combined with p73 exon 2 G4A was proved to be associated with a significant increased risk of squamous cell carcinoma of the head and neck (SCCHN)^37^. *KRAS* is an oncogene which encodes a GDP/GTP-binding protein to regulate cell proliferation and differentiation^38^-^40^, and the relationship between *KRAS* polymorphisms (rs712) and the risk of cancer in the Chinese population has been revealed^41^. Loss of heterozygosity (LOH) and allele-specific expression (ASE) have frequently observed for tumor suppressor genes. For instance, *FGFR2* gene expresses in multiple alternative splicing forms with *FGFR2b* and *FGFR2c* as two major transcripts, but it is transcribed as *FGFR2b* only from one strand (GTA) by mRNA reads at rs1047100^42^, ^43^. Therefore, these mutations in ZJB-ENC1 show its potential value for further biological research.

Additionally, various novel mutations in these genes were detected, which may reveal some new mechanism in cancer. *ARID1A*, as a tumor suppressor, regulates *CDKN1A* and *SMAD3* transcription and tumor growth by collaborating with p53^44^. The *PPP2R1A* gene encodes a constant regulatory subunit of protein phosphatase 2, which is implicated in the negative control of cell growth and division, and its functional mutation (rs11453459) in the promoter contributes to the decreased risk of hepatocellular carcinoma^45^. The catalytic *PIK3CA* and regulatory *PIK3R1* belong to the phosphoinositol-3-kinase (PI3K) family, and the abrogation of these two subunit genes can reduce proliferation, migration, and invasion in cancer cells^46^. *MLH1* repairs DNA mismatch by mediating protein-protein interactions, defects in *MHL1* will lead to the loss of DNA mismatch repair, which is associated with the microsatellite instability (MSI)^47^. *CCNE1*, as an oncogene, determines cell division by regulating the transition from G1 to S phase^48^. Based on the WES results, further functional characterization was required for these novel mutations. Thus, ZJB-ENC1 cells also provide an available tool to get a deeper understanding of tumorigenesis and the development of EC.

## Conclusion

In summary, ZJB-ENC1 cell line was derived from primary tumor tissue of a patient with poorly differentiated endometrioid adenocarcinoma with specific biological characteristics. It was well-established and serially cultured *in vitro* for two years. This cell line could be a good model for the study of cancer biology and targeted-therapy of ENC. The whole exome sequencing of the cells also provides deeper insights into genetic abnormalities in ZJB-ENC1 cells and gives the direction for further EC research. However, owing to the deficiencies of the WES method, the whole genome sequencing with high depth will be taken into consideration in future study to obtain a more comprehensive and detailed genomic characteristic of ZJB-ENC1 cells. Moreover, proteomics should be introduced for more tumor-associated markers.

## Figures and Tables

**Table 1:** Short Tandem Repeat (STR) Analysis of ZJB-ENC1 cell lines

Loci	Allele1	Allele2	Loci	Allele1	Allele2
D5S818	9	10	FGA	22	26
D13S317	10	10	D2S1338	20	21
D7S820	11	12	D21S11	30	30
D16S539	9	12	D18S51	12	17
VWA	17	20	D8S1179	10	10
TH01	9	9	D3S1358	15	15
AMEL	X	X	D6S1043	14	17
TPOX	8	8	PENTAE	14	27
CSF1PO	10	11	D19S433	13	13
D12S391	18	19	PENTAD	9	13

**Table 2:** The distribution of SNPs and InDels of ZJB-ENC1 cells from the whole exome sequencing

Term	Number
Total	83926
downstream	498
exonic	27745
exonic;splicing	42
SNPs and InDels in conding region	27787
synonymous SNV	11648
nonsynonymous SNV	14293
stopgain	710
stoploss	18
nonframeshift insertion	121
nonframeshift deletion	109
frameshift insertion	108
frameshift deletion	123
unknown	657
intergenic	6522
intronic	33270
ncRNA_exonic	3169
ncRNA_exonic;splicing	6
ncRNA_intronic	3453
ncRNA_splicing	25
splicing	233
upstream	698
upstream;downstream	25
UTR3	6542
UTR5	1692
UTR5;UTR3	6

**Table 3:** Mutations of 10 important genes of ZJB-ENC1 cells

Gene	Ref	Alt	Location	avsnp150
TP53	G	A	exonic	rs397516436
	G	T	intronic	rs17883323
	CCCCAGCCCTCCAGGT	-	intronic	rs59758982
ARID1A	C	T	intronic	rs767585532
	C	T	exonic	rs879255270
PPP2R1A	C	A	UTR5	rs535966011
	A	G	intronic	rs755531
	C	A	intronic	rs8100600
	C	A	UTR3	
	-	T	intronic	
PIK3R1	C	T	exonic	rs706713
	G	A	intronic	rs171649
	A	C	intronic	
	G	A	exonic	rs3730089
	C	T	UTR3	rs66666989
	A	C	UTR3	rs78046963
	TG	-	UTR3	rs58984754
	-	TA	UTR3	rs531318827
CTNNB1	G	A	exonic	rs28931589
	G	A	exonic	
	C	A	intronic	rs2691680
	-	TAAT	UTR3	rs16339
PIK3CA	G	A	exonic	rs1057519929
KRAS	A	T	UTR3	rs1137189
	T	C	UTR3	rs4597149
	G	A	UTR3	rs4285970
	A	C	UTR3	rs712
	C	T	exonic	rs4362222
	-	A	UTR3	rs71065923
	-	AA	UTR3	
FGFR2	G	A	intronic	rs2278202
	T	C	exonic	rs1047100
	A	C	intronic	
	A	-	intronic	rs796869588
MLH1	G	A	intronic	rs41562513
CCNE1	C	T	exonic	rs7257694
	C	A	UTR3	rs1406
	CTT	-	intronic	rs569537342
CDKN2A	C	G	UTR3	rs11515
